# The transformative potential of a Global Urban Agenda and its lessons in a time of crisis

**DOI:** 10.1038/s42949-023-00087-z

**Published:** 2023-03-11

**Authors:** Jessica Espey, Susan Parnell, Aromar Revi

**Affiliations:** 1 University of Bristol, School of Geographical Sciences, Bristol, UK; 2 University of Bristol, School of Geographical Sciences and African Centre for Cities, University of Cape Town, Cape Town, South Africa; 3 Indian Institute for Human Settlements, Bangalore, India

**Keywords:** Politics and international relations, Sustainability, Geography, Geography

## Abstract

2015 was a seismic moment for urban stakeholders around the world. A coalition of policymakers, academics and practitioners came together to successfully advocate for an urban goal to be included in the UN Sustainable Development Goal framework. Although the value of a place-based approach to development has been demonstrated by a number of cities and countries worldwide, it was 2020–2022 (three years of cataclysmic global events) that highlighted the necessity for a universal place-based approach to planning in order to foster resilience and sustainability. In this article, three academic-practitioners reflect upon the transformative potential of the 2015–16 urban agendas.

## Introduction

2020–2022 brought into sharp relief a number of complexities facing the first fully urban generation. First, increasing zoonotic diseases and pandemics; second, more disasters, conflict and extreme climatic events; third, the complexity of high-density living and rapid urbanization, fourth and relatedly, exponential demographic growth in some parts of the world, and the migratory trends associated with it; and finally, increasing inequality in incomes and employment, exacerbated by the COVID-19 epidemic^1–3^.

Whilst many city and national leaders have fought valiantly to control COVID-19, to tackle wildfires, manage water shortages, respond to typhoons, war and so much more, what has become explicit in the opening years of this decade is that no one strategy is comprehensively working. The interwoven nature of these challenges and their deeply local ramifications require macro analytical capacity alongside integrated community responses and local, networked action. Particularly pressing is how crises, individual and multiple, are governed as they play out in high-density areas, including the 10,000 small, medium and mega-cities around the world today, home to 55% of the world’s population^4,5^. As noted in the UN Secretary General’s 2020 report on COVID-19 and urbanization, “with an estimated 90 percent of all reported COVID-19 cases, urban areas have become the epicenter of the pandemic”^6^. And whilst the distribution of the epidemic has since changed, this demonstrates the vulnerability of high density, globally connected environments.

The urban agendas, agreed in 2015–16 through two intergovernmental processes, explicitly attempted to tackle the new urban locus of policy-formation and implementation. Whilst both processes had different points of emphasis, they shared a commitment to place-based development, emphasized new forms of devolved governance, the heavy use of multi-scale data for planning, and had at their heart a resilience agenda. Drawing on emerging literature about the place of cities in world crises and auto-ethnography, we trace the emergence of these urban agendas. We consider what made the focus on the urban such an innovative proposal, if and how this commitment to urban development has been implemented, and its potential value as a source of reference in the coming decade as we grapple with governing more people, in new settlements plagued by informality, inequality, poverty, and managing the fresh and compounding crises that are already upon us. Finally, we propose a new, reinvigorated ‘Global Urban Agenda’, which builds on these two foundational intergovernmental processes but goes further to bring more coherence to the concept and the ambitions of sustainable urban development.

We, the authors, are fortunate to have been closely involved in both the 2030 Agenda deliberations and the subsequent Habitat III process. We directly engaged with Member States and other negotiators during the deliberations, both as expert academic advisers, members of the UNSDSN, and as the convenors of the UrbanSDG Campaign, a multi-stakeholder coalition of powerful urban actors, discussed below. Drawing on auto-ethnographic practice, we compiled notes of our experiences and reflections and critically analyzed them to identify common observations about the deliberation process. We have twinned these observations with insights from other published literature so as to help triangulate our findings. We do note however that our experiences and the limited published literature on these processes may not fully account for the experiences of many other urban stakeholders engaged in the process such as secondary city representatives and those not already part of established urban networks.

## The existing urban narrative

From 2015–2016 world leaders agreed upon a vision for urban development articulated during two global summits. The 2030 Agenda for Sustainable Development (the 2030 Agenda) was finalized in September 2015 following three long years of negotiation. The ambition of the 2030 Agenda was to articulate a sustainable development roadmap for the world through a set of common goals and targets (Sustainable Development Goals, or SDGs). These goals would encapsulate the issues embodied by the Millennium Development Goals (MDGs), most notably social wellbeing, but go further to consider other economic and environmental concerns. Crucially, over the course of the negotiation governments recognized the importance of a place-based approach to development, as well as the unique array of challenges facing existing and emerging urban communities, through the inclusion of Sustainable Development Goal 11; a dedicated goal on sustainable cities and human settlements.

The Second Summit was the United Nations Conference on Housing and Sustainable Urban Development (Habitat III), which took place in Quito, Ecuador on 20th October 2016. The Conference resulted in a ‘New Urban Agenda,’ (NUA), which was further endorsed by the UN General Assembly (UNGA) during its seventy-first session on 23rd December 2016. The NUA built upon the outcomes of two previous Habitat conferences, which focused predominantly on housing and service access^7,8^, but also upon the achievements of the urban community during the course of negotiations over the 2030 Agenda.

Both of these international negotiations were seminal for urban stakeholders, including local and regional government representatives, urban academics, community groups and residents. The inclusion of Goal 11 in the SDG agenda was a major coup for urban stakeholders engaging internationally whose only platform for negotiation on global urban development policy had until that point been the Habitat Conferences (in 1976 and 1996)^9^. Habitat I and II had focused attention on cities as infrastructural hubs with immense lending potential^10^, whilst also bringing to the fore issues of urban poverty, access to adequate housing and basic services^11^. Whilst attention to these thematic issues was an achievement, the Habitat conferences did not establish a holistic Global Urban Agenda, nor did they recognize the significance of cities as potential determinants of global ecological integrity^12^ or, notwithstanding powerful boosterish arguments^13^, as sites of global economic activity, job creation, and concentrations of wealth. It was only in the run up to 2015, thanks to the extensive inputs of local government and other urban stakeholders, that the international community acknowledged the central role of sub-national governments and urban policy for determining our pathways towards sustainable development^14–16^. First, the SDGs overturned decades of narrow, sectoral planning by recognizing the concurrent, interconnected social, environmental and economic dimensions of cities, by including explicit targets relating to each dimension; access to housing, transport, green public space and cultural heritage, inclusivity and participation in city planning, sustainable urban growth and land use, minimizing environmental impact, and natural disaster preparedness. Although SDG 11 did not include mention of other important urban concerns like poverty, inequality, and access to services, negotiators repeatedly stressed the interconnectedness of the goals. Indeed, the 2030 Agenda acknowledged in the preamble the necessity for governments to ‘work closely on implementation [of all the goals] with regional and local authorities…’^17^ thereby implying the relevance and applicability of all of the goals at the local level.

A second achievement of the 2030 Agenda and the inclusion of Goal 11, alongside the acknowledgement of place-based or spatial determinants of development, was that it signaled ‘UN members’ acceptance of some form of devolution in governance…’^18^. More specifically, it recognized the necessity for national governments to actively involve, support, and finance local authorities who would be at the forefront of much of the effort to promote sustainable development and stem climate change, specifically noting that ‘sustainable urban development and management are crucial to the quality of life of our people’^19^. Not only was this a win for the SDG process, but for other international processes emerging concurrently such as the Paris Climate Agreement, negotiated concurrent to the SDGs and signed in December 2015, the Sendai Framework for Disaster Risk Reduction, and the ongoing negotiations over the implementation of the Convention on Biological Diversity.

Whilst the 2030 Agenda and its dedicated urban goal recognized ‘a place for cities at the UN-hosted global development policy-makers table’ and highlighted a few central concerns, it did not elaborate on the substance of that agenda beyond a handful of targets^18^. Habitat III was a parallel but complementary 20-year urban dialog, which although lacking in high-level political engagement, provided an international forum within which urban practitioners and policymakers could elaborate on this new urban SDG commitment and provide the beginnings of a normative and operational framework for global urban policy^20^. In particular Habitat III, and its outcome document ‘The New Urban Agenda’ (NUA), helped to articulate five innovative elements, which advanced and complemented the priorities laid out in the SDGs.

These included;

### The Right to the City

The Right to the City is not a new concept, having been first articulated by the sociologist Henri Lefebvre in 1968. Lefebvre described it as an attempt to ‘reclaim’ the city as ‘a co-created space’^21^; creating a habitat for people and life, fostering social interactions and culture, instead of promoting market interests and commodification. Importantly, it is about empowering residents and not designing urban landscapes purely on the basis of market incentives. The concept was actualized into law in Brazil in 2001, where a federal law (Law 10.257) was passed establishing a City Statute, or new legal-urban order, which would aim to provide land access and equity. In particular, it aims to recognize the social function of the city and collective interests over individual ownership rights^22^. During the Habitat III negotiations the term was revitalized and the Brazilian example was cited internationally, identified as a new paradigm that sought to tackle complex urban social challenges whilst also focusing on empowering residents, providing political opportunities, and promoting urban culture^23^. In the NUA it was ultimately described as “a vision of cities for all, referring to the equal use and enjoyment of cities and human settlements, seeking to promote inclusivity and ensure that all inhabitants, of present and future generations, without discrimination of any kind, are able to inhabit and produce just, safe, healthy, accessible, affordable, resilient and sustainable cities and human settlements to foster prosperity and quality of life for all”^24^.

The right to the city paradigm was however deeply contentious, as it borrowed from human rights frameworks as well as certain countries’ conceptualizations of the right to safe and healthy habitats, which were not shared by all. In emphasizing democratic management of the city, the rights-based approach was directly contesting policies like forced slum clearance and other oppressive urban management measures. Fiercely negotiated during the Habitat III proceedings, the language of the right to the city was eventually included, thanks to strong promotion and population mobilization in Latin America and some European countries^25^.

### A focus on equity

A key theme of both the SDG negotiations and Habitat III’s proceedings was a focus on equity. Learning lessons from the pursuit of the Millennium Development Goals, which had often prioritized interventions for the near poor at the expense of the most disadvantaged, both agendas place a strong emphasis on ‘leaving no one behind’^26,27^, and by focusing on spatial inequalities, ‘leaving no place behind’ too. Whilst equity was a cross-cutting theme of the SDGs it was explicitly recognized in SDG 10, which aimed to reduce both vertical and horizontal forms of inequality. In the context of Habitat III, it was noted that cities and urban environments often see much more acute spatial inequalities, which are often masked by aggregated national data^28^. In particular, cities are sites of informal and unregulated employment, in which the majority of urban workers are occupied in low-income countries^29^. Furthermore, cities face unique equity challenges relating to housing and service provision, not least of all for the 1 billion urban people living in informal housing and slum conditions^30^; a challenge reflected in SDG 11’s first target (11.1), which aims to ‘ensure access for all to adequate, safe and affordable housing and basic services and upgrade slums’^31^. The NUA echoes the place-based thrust of the SDGs by specifically noting social and economic exclusion and spatial segregation as ‘an irrefutable reality in cities and human settlements’^32^. To tackle the persistent challenges of inequality, the NUA proposed focusing on the way cities are ‘planned, designed, financed, developed, governed and managed’^33^, rather than proposing sector specific interventions. It did however call on cities to adopt an explicit commitment to tackle gender inequalities, through effective participation, equal leadership opportunities, and equal rights^34^.

### Urban ecology and resilience

In a departure from earlier Habitat positions, a key theme of the Habitat III dialog process and throughout the outcome document is a focus on territorial development, including local ecology and resilience. The link between human settlements, risk and resilience was also explicitly identified in SDG 11—which included targets on disasters planning and preparedness (11.5 and 11.b). A major inspiration for the inclusion of a strong risk narrative in SDG 11 was the IPCC’s published work on urban adaptation and mitigation, specifically Chapter 8 of *Climate Change 2014: Impacts, Adaptation and Vulnerability*, the IPCC Working Group II contribution to the Fifth Assessment Report^35,36^. Key concerns articulated in this report and later reflected in the NUA were preparing cities for more extreme climate-related events, and building adaptive capacity, but doing so through increased resident participation and inclusion. It was noted that this would be particularly important for managing climate-induced human migration, as forced and unregulated movement can often create and perpetuate urban exclusion and unsafe informal settlement^37^. The NUA also committed cities and member states to focus on city resilience and climate adaptation through the development of quality infrastructure (including strengthening and retrofitting all risky housing stock), nature-based solutions, and more careful spatial planning. The critical role of local governance was affirmed by the push for an explicit focus on those in the most risk-prone areas of formal and informal settlements, including slums^38^.

### National Urban Planning including National Urban Policies

A key contribution of the NUA, which took forward the commitment to national and regional development planning articulated in SDG 11 targets a and b, was to strengthen national urban planning frameworks to help cities and local governments fulfill their commitments. Drawing on recommendations of the specially constituted Expert Policy Group, the NUA called specifically for National Urban Policies, which would “empower them [local government leaders] as policymakers and decision makers, ensuring appropriate fiscal, political and administrative decentralization based on the principle of subsidiarity”^39^. National Urban Policies were intended to be a mechanism to set in writing the remit, control, and obligations of local governments, as well as how they would be supported by national governments administratively, legally and financially. As described by UN Habitat, ‘a national urban policy should provide the general framework to orient public interventions in urban areas and be a reference for sectoral ministries and service providers’^40^. An important consideration in these policies is that they should be formed through inclusive and participatory processes, which ensure a dialog between local and national actors on the expectations of both parties. While far from perfect, South African national urban reform initiatives are instructive of the various modes through which multi-scale action and reform can be considered^41,42^.

### A supportive regulatory framework

Related to the commitment for national urban policies, the NUA placed a strong emphasis on the importance of an empowering regulatory environment, which enables cities to effectively manage public services and raise sufficient revenues. In many cities around the world, a major hindrance to effective public service delivery and sustainable development planning is the lack of city authority over key urban services (take for example the governance challenges of the transport systems in the city of New York^43^. A key objective of the NUA was to identify such political power dynamics and make it more feasible for local authorities governing cities and human settlements to be able to make the infrastructural and service decisions required in order to upgrade, retrofit and deliver sustainable and equitable public services. Other crucial regulatory dimensions referred to in the NUA are the regulation of land and security of land tenure within a city (recognizing the benefits of collective use as per *the right to the city*) and the legal and social protections of work and employment^28^, which have been shown to be particularly important in the light of economic recession brought about by the COVID-19 crisis.

For many cities around the world, it is not so much the dominance of federal, state or other influences over urban services, but instead a shambolic network of public, private and informal providers, which cause problems. In such instances, the network of providers may have evolved organically with the rapid and chaotic expansion of the city, for example in Delhi, Kinshasa or Lagos, which have grown in population by an average of 3-5% each year since 2000^44^. In such instances the public sector, and specifically national government, has a crucial role to play in ensuring greater capacity of local governments to regulate such markets and to provide more services or production directly^45^.

Devolution is not only about having the legal and regulatory authority to make independent decisions about services, but about having sufficient resources. Most cities around the world are dependent upon national funding and are not able to raise independent municipal revenues. The NUA commits Member States to address localization through “robust legal and regulatory frameworks for sustainable national and municipal borrowing, on the basis of sustainable debt management, supported by adequate revenues and capacities, by means of local creditworthiness as well as expanded sustainable municipal debt markets when appropriate”^46^. It also encourages drawing upon (and/or establishing) appropriate intermediaries like development banks and pooled financing mechanisms.

## Transformative coalitions

The NUA, agreed at Habitat III, elaborated upon the SDGs, providing important principles and operating procedures to help inform implementation of sustainable development policies at the urban level. However, what was transformative about these two negotiations and the urban vision they articulated was not only the substantive issues identified, but some of the unique processes and coalitions they established;

### A global urban coalition

The global urban community is made up of a wide range of actors; city networks, advocacy coalitions, city leaders and their administrations, academics, and NGOs. Prior to the commencement of the SDG negotiations this epistemic community had been fragmented, unable to cooperate due to their championing of their individual interests, having to compete for space in the international arena and in their ability to fundraise from international donors.

In 2013 the Open Working Group invited a global UN research network, called the Sustainable Development Solutions Network (SDSN), to help convene a dialog on urban issues among the deliberating Member States. It marked a turning point among the global urban community, encouraging the heads of organizations such as SDSN, Cities Alliance, UN Habitat, United Cities and Local Governments and ICLEI to all meet and discuss how they could cooperate and mobilize city representatives to make a concerted case for the inclusion of an urban goal in the future development goals. SDSN and UN Habitat also had the opportunity to provide inputs to the negotiations with written products, such as a position paper on *Why the World Needs an Urban Sustainable Development Goal*, which was authored in partnership with leading urban networks^14^ and included a proposal for an urban goal, which contained many of the core elements agreed to in the final formulation of SDG 11.

The result of this cooperation was the Urban SDG Campaign (http://urbansdg.org/), a global campaign of hundreds of local governments and world leading urban networks and institutions, including C-40, Cities Alliance, Communitas, ICLEI, Metropolis, Slum Dwellers International, SDSN, UCLG, UN-Habitat, and World Urban Campaign. The campaign aimed to bring actors together to advocate for and design an urban SDG, including its targets and indicators. Crucial contributors to the campaign were groups like SDSN and Communitas, working with UN-Habitat, who worked to mobilize supportive Member State representatives in New York, such as the Latin American group, and to provide them with useful inputs for their deliberations^47^. Even though there were some challenges within the coalition (for example a lack of grassroots representation) the contribution of this rather ad hoc effort was considerable; “Even before the SDGs were finalized, mayors and local leaders successfully pushed for a dedicated goal to “make cities inclusive, safe and resilient and sustainable” (goal 11) through the global Campaign for an Urban SDG”^48^.

### A strong science-policy interface

A unique attribute of the UrbanSDG campaign was that it was not just made up of advocates and city representatives. Scientists and urban academics were also actively involved as members, helping to develop the group’s positions and policy outputs. So much so that the campaign provided the “key intellectual energy behind the push for an urban SDG”^7^. Key organizations represented in the coalition included among others the Columbia University, the Indian Institute of Human Settlements, the University of Pennsylvania, the Stockholm Resilience Centre, the University of Gothenburg, the University of Cape Town, International Institute for Environment and Development (IIED), EThekwini Municipality, South Africa, New School University, UN Habitat and the UN SDSN. Actors from these organizations had been writing extensively on urban planning and sustainability for decades prior to the negotiations commencement. They also had both personal academic and designated roles in the multilateral process, for example Genie Birch from the University of Pennsylvania was the Chair of the General Assembly of Partners (GAP) for Habitat 111, Susan Parnell was closely associated with ISC (International Science Council) who are the formal voice of UN’s Scientific and Technical Major Group, and Aromar Revi and David Satterthwaithe were coordinating lead authors of the IPCC’s Working Group II report on Climate Change Adaptation. The coalition worked together to prepare common position papers and statements, working papers and reports, and held a series of important convenings in London, Gothenburg, Bellagio and Bangalore to bring together key urban constituents and forge common, evidence-based proposals.

Nevertheless, it was not until after the dust had settled on the SDG negotiations that academic literature on the urban goal was widely published^49–52^, reflecting the real-time and often reactive engagement between scientists and policymakers during both negotiation processes.

### Active engagement of city stakeholders

A defining feature of the Urban SDG Campaign was the active engagement of more than 300 city and local government leaders. Although local governments have a representative seat in the UNGA through the “Major Groups” (9 representative seats for sectors of civil society to participate in UNGA proceedings), it is an observer seat shared by thousands of local government actors, under the coordination of United Cities and Local Governments. The Urban SDG Campaign, working closely with its city-representative members, helped to bring more city voices to the Member State deliberations, catalyzing “an ongoing movement to strengthen the voice of local leaders in the SDGs monitoring process, led by global city networks, such as United Cities and Local Governments, the Global Taskforce of Local and Regional Governments, and ICLEI Local Governments for Sustainability”^48^. The beginning of this movement was the UCLG World Congress convening in Rabat, where plenary members discussed the need for a Global Urban SDG Campaign, helping to mobilize local government engagement and support. It was given further momentum by a conference convened by SDSN with the Pontifical Academy of Sciences in Vatican City and which included Pope Francis’s participation^53,54^. Governor Jerry Brown voiced the most compelling call for local government action and engagement in Paris and the SDGs when he paraphrased Marx, saying cities ‘have nothing to lose but their chains’^54^.

By the time of the Habitat III conference, in 2016, UCLG, ICLEI, C40 and various other local government networks had mobilized over 2000 representatives of local and regional government to attend the conference^55^, a sizeable increase on the 500+ Mayors who participated in Habitat II and the parallel First World Assembly of Cities and Local Authorities (WACLA). The participation of so many local government officials reaffirmed the eagerness and the relevance of their participation in Member State deliberation processes. It also marks the de facto incorporation of cities into the heart of the multilateral urban agenda setting processes.

## Implementation and use since 2015/16

Since 2015 there has been a flurry of activity relating to implementation of the SDGs and the New Urban Agenda. The most promising efforts pertain to localization, voluntary local reporting to the UN High-Level Political Forum (the annual accountability forum for the SDGs) and the increased use of data to improve local sustainable development policy-formation.

### Localization

Since the SDGs were adopted there has been an effort to integrate them into national policy frameworks and to coordinate SDG implementation through national coordination mechanisms at the highest levels of government. For example, Bangladesh has aligned its national strategy—Vision 2041—to the SDGs, and according to a 2020 review a further 27 countries have either developed a national SDG strategy or have integrated the SDGs into their sectoral action plans^56^. Now that countries have established national SDG implementation mechanisms more and more attention is also turning to localization of the goals; “the process of taking into account sub-national contexts in the achievement of the 2030 Agenda”, and how local policy can be adapted to support the Goals^49,57^. For example, in Argentina, the national SDG strategy contains a chapter dedicated to localization with an emphasis on implementation at the provincial level^58^. In Bangladesh, the Voluntary National Review (which is a country’s SDG progress report) includes a section devoted to local SDG implementation processes, focused at the district and upazila or subdistrict level^58^. Whilst in the City of eThekwini, South Africa efforts have been made to align the city’s Integrated Development Plan to the SDGs^59^. According to UCLG’s 2020 annual progress report “more and more, LGAs and LRGS (Local Government Authorities and Local Regional Governments] are mainstreaming the SDGs into their policies and plans. Hundreds of cities have embedded the SDGs in their local strategies and medium-term planning objectives”^58^.

### Voluntary local reviews

A key part of the localization of the 2030 Agenda is place-based reporting on SDG implementation progress. The High-Level Political Forum invites Member States to report annually through Voluntary National Reviews but since 2017 there has been a movement among local government leaders to complement this national reporting with Voluntary Local Reviews. The two Local and Regional Governments Forums organized in 2018 and 2019 have particularly catalyzed this conversation, highlighting VLRS as an opportunity for local information sourcing and peer to peer city exchange. In 2018 New York City and three Japanese Cities were the first to publish VLRs, but as of 2021 there have been more than 160 VLRs released^60^. Furthermore, 6 pilot countries are being supported by UCLG (Costa Rica, Ecuador, Benin, Mozambique, Kenya, and Nepal) to develop Voluntary Sub-national Reviews on the current state of SDG localization^61^. Also in 2021, for the first time in five years, many LRGs were invited to participate in VNR preparation in more than half (55%) of the countries submitting annual reports^58^.

### Data-based approaches

A key attribute of the SDG and Habitat III agendas is their focus on data and using evidence to guide policy. As stated in Declaration of the 2030 Agenda, “quality, accessible, timely and reliable disaggregated data will be needed to help with the measurement of progress and to ensure that no one is left behind. Such data is key to decision-making”^62^. National Governments have recognized this through their use of indicators and quantitative progress tracking, but an interesting development is how many local and regional governments are also pursuing a data-based approach to support local sustainable development. Between 2016-2018 SDSN’s thematic research network on data (TReNDS) documented SDG implementation approaches in eight cities and regions worldwide, most explicitly through their Local Data Action Solutions Initiative. They identified eight cities and regions pursing data-based SDG implementation albeit in very different ways. In Aruba for example the island was focused on developing integrated data systems that could track policy impacts in marine and land areas simultaneously; in Brazil a process was set about to develop more than 50 highly contextualized local development indicators to aid local SDG progress; in Colombia and India cities were focused on data communications and visualizations to make the cities’ progress more visible, accessible and accountable to residents; whilst in San Jose, California, a process was developed to align the city’s existing indicators with the SDG indicators and find a hybrid reporting mechanism that would work for both ends^63–65^. Concurrently a number of research institutions have worked with governments to prepare Urban SDG Indices, to help local governments take stock of their baselines, progress and challenges towards SDG implementation^66^. Although the approaches vary considerably, common to all regions studies was a sense that “data and indicators provide a common language for coordinating sustainability efforts”^67^.

## Limitations of the existing urban narrative

In spite of the important issues raised by SDG 11 and the principles and operating procedures that the NUA further elaborated, both agendas have been subject to critique. Some critics point out that the agendas fail to tackle important drivers of inequality and urban degradation^68^ or means of implementation such as the nature of devolution and local financing^69,70^, whilst others express concern about the reductionism of these agendas and their strong emphasis on quantification^71^. Five particular deficiencies or limitations are discussed below. These do not negate the utility of the agendas overall or their value as a framework for inserting urban issues into international processes but are areas in need of further consideration. Only by doing so can a reinvigorated ‘Global Urban Agenda’ be established, which can align countries and local government behind a comprehensive and solutions-oriented strategy for urban sustainable development.

### Intra-urban inequality

Whilst widening global inequality is a well-recognized phenomenon^72,73^, acute and growing intra-urban inequalities are also a pressing concern, particularly in the face of rapid urbanization, and yet are severely under-represented and discussed in international policy frameworks. Intra-urban inequalities are characterized by concentrations of disadvantage often focused on particular social groups; “people sharing common characteristics are often found in close proximity to each other, and at the same time, separated from other social groups. Such a separation is also known as spatial segregation”^74,75^. A 2018 study by the OECD found that income inequality, migrant status and public transport systems were particularly crucial to understanding growing urban inequalities and that the “concentration of lower-income and minority groups is deemed particularly problematic when it leads to worse economic outcomes. Evidence from cities in the Netherlands shows that a 1% increase in the share of migrants is associated with a 0.32% increase in the share of poverty”^76^. The fact that there are clear observable drivers of intra-urban inequality, such as migration patterns, lays it open to clear policy intervention and action and yet the GUA fails to give the topic due consideration.

Whilst global economic and social inequality is a core theme of the SDGs (best encapsulated in Goal 10 which aims to reduce inequality within and between countries), the agenda fails to highlight the pernicious effects of spatial inequalities. Although SDG 11 implicitly acknowledges a distinction between urban and rural, spatial inequality is not listed as a core consideration within target 10.2 (“By 2030, empower and promote the social, economic and political inclusion of all, irrespective of age, sex, disability, race, ethnicity, origin, religion or economic or other status”)^77^. Furthermore, whilst SDG 11 does consider important manifestations of urban inequalities, such as access to green spaces, accessible transit systems, and inclusive planning mechanisms, there is no explicit recognition of income segregation and how space can affect forms of economic and social deprivation^74^. Relatedly, missing from both agendas is a recognition of the importance of space and design, and the impact of this design can have upon service delivery, social cohesion and inclusion^74^.

### Territoriality

Somewhat related to issues of spatial inequality is the concept of territoriality. Territoriality is a term used in urban studies to delineate how people use urban space; their ownership, occupancy rights, and interactions with their communities^78^. Above all else it is about using territorial or spatial frameworks to understand social and economic interaction. Whilst the GUA does provide a macro-framework for considering urban dimensions, it fails to provide a holistic approach to meeting social, economic and ecological dimensions of sustainability development within one place. The concept of territoriality proposes engaging with the apparent contradictions of informality, community, locality and the principles of social, economic, environmental/climate and epistemic justice all in governance frame. The need for such an approach has been laid bare by the COVID-19 epidemic, which has highlighted the necessity to consider how people use urban space for their social and economic needs, whilst also interacting with their built and natural environments.

### High-level political engagement

Whilst the SDGs and the accompanying 2030 Agenda were endorsed by 193 Heads of State and Government, signaling strong support for or at least acceptance of the urban goal, Head of State participation at Habitat III was minimal. Only 4 Heads of State, including the host (H.E. Mr. Rafael Correa Delgado, President of the Republic of Ecuador) attended the event. Some contend that this was merely a reflection of post-summit exhaustion and “little diplomatic energy left”, following the SDGs and Paris Climate Agreement negotiations the preceding year^79,80^, however, others suggest this reflects a lack of serious national engagement with the NUA. In spite of 167 countries formally approving the New Urban Agenda, only 5 national governments put forward voluntary national implementation plans^80^. Furthermore, neither former UN Secretary-General Ban Ki Moon nor current Secretary-General António Guterres have said much publicly about the NUA since its adoption, and when they have it has been to reinforce its relevance as a means of implementation for the SDGs, not as an important supplementary policy document; “Secretary-General Guterres views the New Urban Agenda as a key component of the implementation of the 2030 Agenda, given that urbanization is a significant influencer in the pattern of development of a country,” according to U. N. spokesperson Stephane Dujarric^80^.

A further, related critique is that in spite of approximately 2000 Mayors and local government leaders attending Habitat III, the outcome document was not written in such a way as to be relevant and accessible for local leaders and officials, making it hard to use thereafter. According to Pamela O’Connor, a councilmember in Santa Monica, California, and the town’s former mayor, “diplomats at the U.N. don’t do any favors with their opaque processes and the bureaucratic language they use…. core Habitat III issues such as affordable housing and equity are discussed constantly at the local level in the U.S., but using different terms and reference points”^81^.

### Productivity

A significant critique of the SDGs and the subsequent New Urban Agenda is their insufficient attention to the means of implementation^69^. Whilst the SDGs raised important substantive issues, they do not address fundamental questions of local government productivity, resourcing and authority. The intention of many was for these issues to be tackled head on in Quito the following year but with only limited high-level participation in Quito the NUA could only go so far, making some commitments on regulatory improvements but without the accountability procedures to see them followed through.

A key objective for many urbanists during the SDG negotiations was to change existing political tendencies to “resolutely ignore[d] urban areas as the loci of the production of 70 percent of GDP in most countries and as the spaces where inequality, unemployment and social conflict are most evident”^82^. As such some early proposals for an urban SDG called for a specific focus upon urban productivity and employment, not only to recognize cities’ influence, power and authority in macro-economic processes but also specific employment challenges relating to informality and workers’ rights^14^. Over the course of the Open Working Group negotiations, however, productivity was moved out of Goal 11 so economic concerns might be tackled within one goal; Goal 8^83^. Whilst having discreet thematic goals provided a simplicity and clarity to the final agenda, the importance of working intersectorally (a point repeatedly stressed in the deliberations) was left to the preambular narrative. The net result of this (as now demonstrated by numerous case studies of health and climate change, mental health and environmental degradation^84^ is that countries have not been incentivized to create inter-sectoral planning and implementation structures. And indeed, without a spatial lens made explicit within the economic goals, the agenda misses a focus on informality of both housing and employment and the rights that are required to support the most vulnerable living and working in informal conditions. One of the disadvantages of silo-ing the discussion of economic activity within one thematic goal was that without a spatial lens and an explicit focus on urban spaces the agenda missed a focus on informality, of both housing and employment.

### Financing

Furthermore, many argued that as economic and employment epicenters, cities should be entitled to more municipal financing, both from central government but also from innovative mechanisms like municipal bonds, evolving loan funds, energy performance contracting and so on. Whilst the topic was infrequently discussed during the 2030 Agenda deliberations, it became a major focus for many advocacy groups in the run up to the Habitat III conference (highlighted at many precursor and side events). Whilst the NUA did commit to fiscal decentralization and strengthening local government capacities to raise revenues, there was however no elaboration on how this should be done and “how this will translate into practice, in different context, remains to be seen”^85^. Furthermore, many of the financing solutions that were discussed at Habitat III focused on “user-end tariffs for services such as water, electricity and sanitation, making city budgets more “efficient” and digitalizing public sector jobs, as well as raising private capital. However, this is unlikely to foster the inclusiveness that is supposed to underpin the Agenda, critics argue”^86^. Particularly important for such innovative financial practices is the legal and regulatory framework of countries, and whether and how authority and powers are granted to local governments to raise independent revenues. As was noted by one leading urbanist in the run up to Habitat III, “trends since the 1990s point towards a greater delegation of responsibilities to local levels of government that are unmatched by the corresponding authority to plan, design, invest and pay for the needed infrastructure”^87^. Whilst the Habitat dialogs raised awareness of this tension, the outcome agreement failed to commit governments to rectify this contradiction. Instead such pivotal outstanding issues were relegated to national dialogs with the hope that these would be clarified through new national urban policies (NUPs).

## Reigniting a Global Urban Agenda to grapple with twenty-first century challenges

2020 alone brought about an unprecedented global pandemic, intense global wildfires, which burned more than thirteen million acres in Australia and two million acres in California^88,89^, increased flooding^90^, and the hottest temperatures this century^91^. The risks and impacts of these anthropomorphic events are particularly intimidating in high-density cities. In the case of the COVID-19 pandemic, for example, cities were the central nodes of the disease’s spread in many countries^92^. As a result, in almost every urban central business district worldwide we’ve seen a catastrophic drop in footfall, resulting in abandoned properties, folded businesses, and deserted public transit systems. We’ve also witnessed a revolution in working models, with 57% of employed people in London, for example, working remotely from home during the first “Lockdown” from February to April 2020^93^.

But in spite of risks that COVID-19 has highlighted with regards to high-density living, the twenty-first century urbanization trend continues. According to the UN’s latest World Population Prospects, “the medium-variant projection [of population growth] indicates that the global population could grow to around 8.5 billion in 2030, 9.7 billion in 2050, and 10.9 billion in 2100”^94^, and “urban areas are expected to absorb virtually all of the future growth of the world’s population”^94^.

According to Ian Klaus, a senior fellow at the Chicago Council on Urban Affairs, the result of the pandemic will not be a move away from cities but instead a fundamental rethink in the way cities are designed; “we are, at this moment, probably prone to overthinking a pandemic’s influence [on the built environment]”^95^. As Klaus (2020), Eltarabily and Elghezanwy (2020) and others have argued throughout 2020, pandemics do not fundamentally limit urbanization but instead affect the way cities are designed, for example encouraging city planners to provide better access to green open spaces, bike lines and pedestrian access^96,97^. “It’s a story of acceleration and continuity rather than one of super profound disruption or revolution. It’s much more about the acceleration or deceleration of trends”^96^. Nevertheless, many have stressed the impending humanitarian crisis that is unfolding in high-density areas with informal settlements, where maintaining personal space and minimizing pandemic transmission will be nigh on impossible^98,99^.

A new governance approach is needed to tackle the impending urban humanitarian crisis and ensure new forms of healthy and sustainable urban living. A new Global Urban Agenda (GUA), which takes the best of the NUA and the SDGs and responds to their criticisms, could have immense value both for national spatial planning and for motivating and coordinating local action. Furthermore, the urban dimensions of many of this century’s challenges—made explicit in the last year—necessitate greater political engagement with urban issues than ever before.

The Global Urban Agenda should have at its core five principles, which build upon and advance elements of the NUA and 2030 Agenda, whilst offering practical guidance for policy-makers looking at emergency, disaster and disease preparedness in the decade ahead:

- 1. The foremost principle of a new GUA should be to move away from sectoral planning and to adopt a spatial or place-based approach to planning and development; looking at the intersecting needs of different geographies and overlaying needs, vulnerabilities and strengths before administering services, planning infrastructure, and so on. A good example of this place-based approach to planning is provided by the city of Los Angeles, which moved quickly to establish a COVID-19 dashboard in the early stages of the pandemic and has since expanded it be a ‘Recovery Dashboard’. This dashboard does not only map cases and death rates across the city, it also maps other key community vulnerabilities such as the location of care homes to encourage advanced preparation and service provision: https://corona-virus.la/los-angeles-recovery-dashboard.
- 2. A crucial principle of the GUA should be a focus on local capacity for urban resilience. The majority of our emergency response, climate response, and planning requirements are local. Councils worldwide are the ones managing lockdowns, outreach, community support, policing, fire service provision and so on. As such, a focus on upon local resilience and capacity is paramount, particularly in high-density urban areas. This may mean investing in building community capacities and informal services where public provision is deficient, for example community health extension worker programs and rallying community health volunteers^100^.
- 3. Governments at all levels need to better engage communities in urban planning and spatial management, which not only fosters civic pride but provides extra capacity for the administration of the community. A good example of this is provided by the city of Medellin, Colombia, where a policy of “Ciclovía” has been introduced. This is the closure of various city streets each Sunday to allow for biking, walking, and other recreational activities. “These events have helped to raise awareness of the negative impact that car traffic has on people’s lives and have been a key part of the city’s ongoing effort to regain street space for pedestrians and bicycles.” It is believed they are also helping to foster safer communities and to give better access to open spaces for all of the city’s residents^101^.
- 4. The GUA and its foundational documents—the 2030 Agenda and its 17 SDGs and the NUA—prioritize equity and particularly the commitment to leave no one and no place behind. This means focusing on the poorest, most vulnerable and hardest to reach first and planning targeted interventions (including spatial interventions) and responses that cater to their needs first and foremost. To enable this a key ingredient is high-quality data. Disaggregated data on population groups, exposures, risks and overlapping vulnerabilities is essential to understand where the most vulnerable are and how best to respond to their needs.
- 5. Now more than ever we need to put science at the forefront of decision-making, learning from the wide evidence inputs that informed the 2030 Agenda and NUA (see for example the preparatory documents prepared for Habitat3.org), as well as the pivotal role that science has played in helping governments respond to COVID-19^102^. The transboundary nature of twenty-first century challenges means that politics alone is not sufficient. Collective knowledge accumulation and scientific study is crucial to understand the drivers of environmental and health change and to design appropriate policy interventions. Curating scientific advisory boards and knowledge networks at all levels of government will be fundamental to an evidence-informed and appropriate response to all aspects of future policy-making, urban or otherwise.

## Conclusion

Whilst global governance is key for managing transboundary challenges, the future of sustainable planning and emergency response is local and regional. For the majority of the world’s residents, local means urban. Adaptation will be crucial to sustainable urban living in the future and a new Global Urban Agenda could offer guiding principles to inform this transformation. As demonstrated by cities like Los Angeles, Medellin, New York, Bristol, Nairobi and others, including many of the world’s growing secondary cities, using a place-based approach to development planning can drive innovation. It necessarily requires multisectoral and multi-scalar collaboration, and when overlaid with the use of high-quality, disaggregated data, capacity building and civic outreach, it can help to foster inclusive, resilient communities, well equipped and empowered to develop local responses to complex economic, social and environmental challenges. A new Global Urban Agenda (GUA), must build on the NUA and the SDGs and respond to their criticisms to help motivate and coordinate local action. Furthermore, this reinvigorated agenda must unify the urban community and bring coherence to a global urban narrative to help foster greater political engagement with urban issues than ever before.

### Reporting summary

Further information on research design is available in the Nature Research Reporting Summary linked to this article.

## Supplementary information

Reporting Summary

## Data Availability

No datasets were generated for the production of this article.
